# Whole-Genome Sequence of *Enterobacter* sp. Strain MF024, Isolated from Soil in Shanghai, China

**DOI:** 10.1128/MRA.00650-19

**Published:** 2019-09-12

**Authors:** Shaofeng Rong, Jin Wu, Qianqian Li, Shuo Zhang, Baoguo Cai, Shimin Guan

**Affiliations:** a School of Perfume and Aroma Technology, Shanghai Institute of Technology, Shanghai, China; Broad Institute

## Abstract

We report here the draft genome sequence of *Enterobacter* sp. strain MF024, a bacterium that can biosynthesize 2-phenylethanol through both the Ehrlich pathway and a *de novo* pathway. It has potential use for the production of 2-phenylethanol.

## ANNOUNCEMENT

*Enterobacter* bacteria are widely found in nature and are a part of the normal bacterial flora of the human intestine (1). In this study, soil samples were collected from Gulf Forest Park in Shanghai, China. Then, bacterial strains were preliminarily screened for growth on Luria-Bertani medium with 2.0 g/liter 2-phenylethanol. *Enterobacter* sp. strain MF024 was further isolated by a 2-phenylethanol detection method (2). This strain showed high 2-phenylethanol stress tolerance and had the ability to biosynthesize 2-phenylethanol. Sequencing of the 16S rRNA gene (GenBank accession no. MG554655) showed 99.0% shared similarity with those of *Enterobacter* spp.

DNA was extracted and purified from a single colony in Luria-Bertani culture using a rapid bacterial genomic DNA isolation kit (Sangon Biotech, China). A sequencing library was built with the Nextera XT DNA library preparation kit (Illumina, USA). The whole-genome sequencing of *Enterobacter* sp. strain MF024 was performed using Illumina MiSeq sequencing technology with paired-end and barcode strategies according to the manufacturer’s instruction (Illumina). Raw FASTQ files were quality filtered by Trimmomatic version 0.36 with the parameters LEADING:3, TRAILING:3, SLIDINGWINDOW:4:15, and MINLEN:36. *De novo* genome assembly was carried out with SOAPdenovo version 2.04 (3). The default parameters were used. The genome coverage was 278.84×, and 26 scaffolds were obtained. The total length of the genome is 4,278,063 bp, with a GC content of 56.69% and an *N*_50_ value of 577,909 bp.

Gene annotation and data analysis were carried out on the free online Majorbio I-Sanger cloud platform (www.i-sanger.com) using Blast2Go version 2.5 and the nonredundant (NR) (ftp://ftp.ncbi.nlm.nih.gov/blast/db/), Clusters of Orthologous Groups (COG) (4), Pfam (v31.0), Swiss-Prot (5), and KEGG databases (6). A total of 4,512 coding sequences (CDS), 2 rRNA genes, and 78 tRNA genes (7) were identified. No plasmids were detected by PlasmidFinder version 1.3 using default settings (8).

The amino acid metabolism and carbohydrate metabolism pathways in this strain were further analyzed. Six genes coding for transaminase (*aspC*, *hisC*, and *tyrB*), phenylpyruvate decarboxylase (*pdc*), and alcohol dehydrogenase (*adhP* and *adhE*) were detected. These enzymes may participate in the Ehrlich pathway to synthesize 2-phenylethanol from l-phenylalanine (9). A total of 10 genes, namely, *aroF*, *aroB*, *aroQ*, *aroE*, *quiA*, *aroK*, *aroA*, *aroC*, *pheA*, and *tryA*, were identified. These genes encode enzymes involved in the pentose phosphate pathway, glycolytic pathway, and shikimic pathway (10). They also participate in a *de novo* 2-phenylethanol biosynthesis pathway. This pathway uses glucose as the carbon source to produce 2-phenylethanol without adding l-phenylalanine (11). Identification of genes involved in the Ehrlich and *de novo* 2-phenylethanol pathways suggests that this strain may have potential use for the production of 2-phenylethanol.

### Data availability.

The whole-genome sequence described here has been deposited in DDBJ/ENA/GenBank under accession no. VCBB00000000. The version described in this paper is the first version, VCBB01000000. Raw sequence data have been deposited under Sequence Read Archive accession no. PRJNA527374. The 16S rRNA gene has been deposited in GenBank under accession no. MG554655.
